# Hearing and Cognitively Impaired Life Expectancies in the United States

**DOI:** 10.1093/geroni/igaa057.1565

**Published:** 2020-12-16

**Authors:** Jessica West, Scott Lynch

**Affiliations:** Duke University, Durham, North Carolina, United States

## Abstract

As the population ages, increased prevalence of cognitive and sensory impairments may pose growing public health challenges. Among the nine modifiable risk factors for dementia, the highest percentage (9%) of dementia cases are attributed to hearing impairment. While much research has examined the relationship between hearing impairment and cognition, almost none has translated these relationships into a meaningful, life course metric: how many years of life individuals can expect to live with both impairments and how hearing impairment affects years lived with cognitive impairment. Our study fills this gap by using Bayesian multistate life table methods applied to nine waves of the Health and Retirement Study (1998-2014) to estimate years of life to be spent (1) with/without hearing and cognitive impairment, and (2) with/without cognitive impairment, conditional on having versus not having hearing impairment. Preliminary results for aim 1 reveal that at age 50, individuals will live 18.9 (18.7-19.2) years healthy, 4.3 (4.2-4.5) years hearing impaired but cognitively intact, 4.2 (4.0-4.3) years hearing unimpaired but cognitively impaired, and 2.3 (2.2-2.6) years with both impairments. Women will spend more years healthy, hearing unimpaired but cognitively impaired, or with both impairments; men will spend more years hearing impaired but cognitively intact. People with more education will spend more years hearing impaired but cognitively intact; people with less education will spend more years hearing unimpaired but cognitively impaired or with both impairments. Our study is one of the first to investigate the implications of hearing impairment for years of cognitively impaired life.

